# [Retracted] Stanniocalcin 2 expression predicts poor prognosis of hepatocellular carcinoma

**DOI:** 10.3892/ol.2024.14847

**Published:** 2024-12-17

**Authors:** Zhen-Hai Zhang, Ya-Guang Wu, Cheng-Kun Qin, Zhong-Hou Rong, Zhong-Xue Su, Guo-Zhe Xian

Oncol Lett 8: 2160–2164, 2014; DOI: 10.3892/ol.2014.2520

Following the publication of this paper, it was drawn to the Editor's attention by a concerned reader that the immunohistochemical data shown in Fig. 2 on p. 2162 had already appeared in a previously published article in the journal *PLoS One.* Upon performing an independent analysis of the data in the Editorial Office, it was subsequently discovered that the data shown in Fig. 1 had also appeared in a (different) previously published article. Owing to the fact that the contentious data in the above article had already been published prior to its submission to *Oncology Letters*, the Editor has decided that this paper should be retracted from the Journal. After having been in contact with the authors, they accepted the decision to retract the paper. The Editor apologizes to the readership for any inconvenience caused.

